# Factors Affecting Post-Stroke Depression in Acute Ischemic Stroke Patients after 3 Months

**DOI:** 10.3390/jpm11111178

**Published:** 2021-11-11

**Authors:** Chan-Hyuk Lee, Su Hong Jeon, Min Ju Kim, Gyu Dam Ra, Yong-Hyun Lee, Seung Hyeon Hong, Byoung-Soo Shin, Hyun Goo Kang

**Affiliations:** 1 Department of Neurology, Jeonbuk National University Medical School, Jeonju 54907, Korea; bluewave0210@gmail.com (C.-H.L.); sbsoo@jbnu.ac.kr (B.-S.S.); 2 Department of Neurology and Research Institute of Clinical Medicine, Jeonbuk National University, Jeonju 54907, Korea; 3 Jeonbuk National University Medical School, Jeonju 54907, Korea; tnghd3677@naver.com (S.H.J.); kmj1127@jbnu.ac.kr (M.J.K.); gyudamra@gmail.com (G.D.R.); ynh376@naver.com (Y.-H.L.); sherry3456@naver.com (S.H.H.)

**Keywords:** Hamilton Depression Rating Scale, ischemic stroke, post-stroke depression, prognosis

## Abstract

Post-stroke depression (PSD) affects approximately one-third of stroke patients. PSD not only impairs recovery and lowers quality of life, but has also serious neurological consequences, high mortality, and stroke recurrence risks. Studies on PSD-related prognostic factors are still lacking, especially environmental factors. Moreover, relieving factors after PSD in stroke patients has not been reported. This study aimed to investigate (study design 1) risk factors for PSD diagnosis after three months, and (study design 2) related factors for the relieving of early PSD after three months. This retrospective study included 227 patients hospitalized for acute ischemic stroke within three days at Jeonbuk National University Hospital from January to December 2019. The depressive status was assessed using the Hamilton Depression Rating Scale (HDRS) at admission and after three months. Clinical and laboratory data were analyzed for relevant prognostic factors. (Study design 1) HDRS score at admission (adjusted odds ratio (aOR) 1.22, 95% confidence interval (CI) 1.14–1.31; *p* < 0.001) and hospitalization period (aOR 1.11, 95% CI 1.02–1.20; *p* = 0.013) were confirmed as prognostic factors of PSD after three months. (Study design 2) The National Institute of Health Stroke Scale (NIHSS) score at discharge (aOR 0.80, 95% CI 0.68–0.94; *p* = 0.006) and HDRS score at admission (aOR 0.80, 95% CI 0.71–0.89; *p* < 0.001) were confirmed as prognostic factors of depression improvement after three months. In conclusion, environmental factors such as hospitalization period could be important in managing PSD. Factors related to PSD improvement are expected to be helpful in establishing a strategy for PSD recovery.

## 1. Introduction

Stroke is a major cause of chronic adult disability and has a long recovery process. Moreover, it is the second leading cause of death worldwide [1]. Approximately 6 million people die from stroke globally every year, while 5.5 million are severely disabled by it [2]. Furthermore, the socioeconomic loss caused by stroke is enormous because the annual medical cost for treating one stroke patient is substantially high [2]. Post-stroke depression (PSD)—a representative complication of stroke—is one of the common psychiatric disorders occurring after stroke [3].

PSD is a mood disorder causing depression and anhedonia that occurs in 18–30% of all stroke survivors; however, only approximately 5% of them are diagnosed with PSD and are treated appropriately [4]. Patients diagnosed with PSD tend to feel less happy, feel less interest and pleasure, experience decreased vitality and fatigue, and suffer from suicidal thoughts. Moreover, they often experience weight loss, insomnia, poor appetite, anxiety, and poor concentration [5]. These psychological and physical symptoms accompanying PSD impair the patients’ cognitive functions and worsen the neurological prognosis. It has been reported that PSD doubles the recurrence rate of stroke within 1 year from the first attack [2]. Thus, it is evident that PSD can delay the recovery of stroke survivors and adversely affect their quality of life. It also interferes with the return of stroke survivors to the community and increases the burden on caregivers [6]. Consequently, it is important to diagnose and treat high-risk patients appropriately, managing in particular the factors that could possibly worsen PSD. 

Although PSD greatly affects the prognosis of stroke survivors and is a common stroke complication (approximately 30% prevalence), only a limited number of patients are treated in clinical practice [4]. Several studies have evaluated the pathophysiology, treatment, and prognosis of PSD. Particularly, several risk factors related to PSD were proposed, mainly focusing on clinical and laboratory factors such as Barthel index, female sex, diabetes mellitus, smoking, and white matter hyperintensity [7,8,9,10]. However, the psychiatric problem is the result of the complex action of not only the patient factors but also environmental factors. Previously, there was a report that it is associated with PSD in cases of living alone [10], but more studies are needed. In addition, previous reports mainly focused on factors affecting the prevalence of PSD. However, there have been few studies about the factors related to the improvement of PSD caused by stroke.

Therefore, this study compared and analyzed test results of patients with acute ischemic stroke during admission and after three months in order to identify factors affecting the occurrence and improvement of PSD, including environmental factors.

## 2. Materials and Methods

### 2.1. Patient Selection

**Figure 1:**
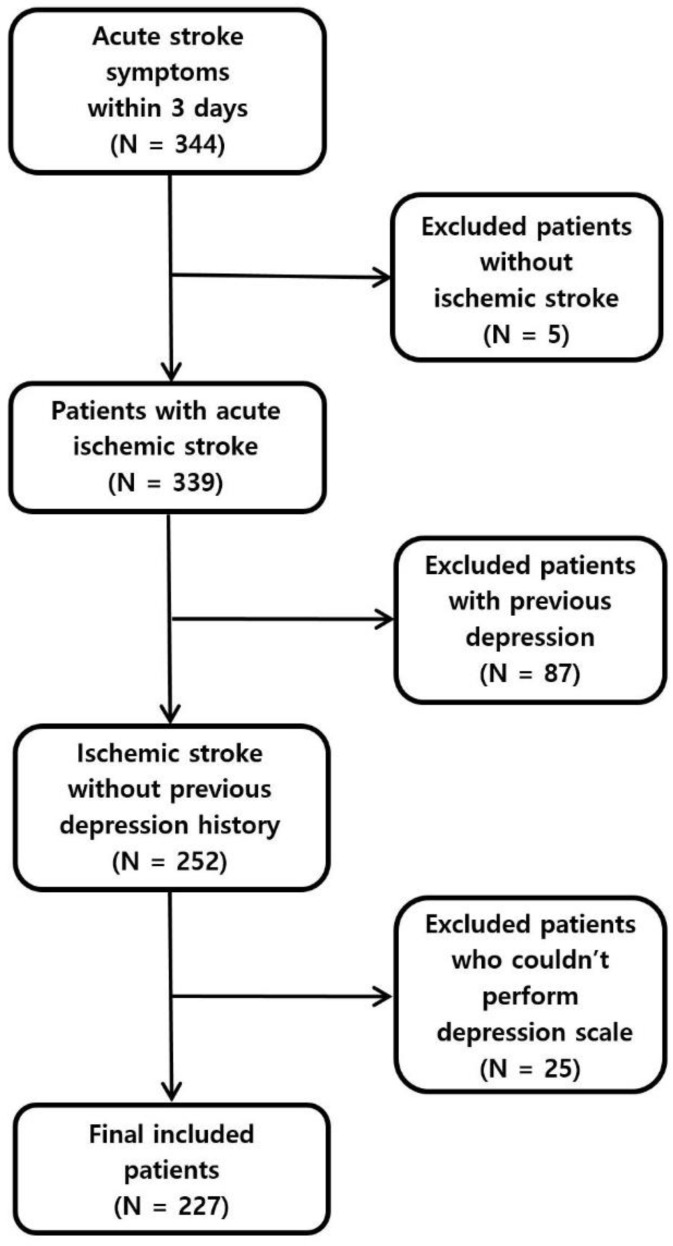
Flowchart of Patient Selection.

This is a retrospective study of 344 patients who visited Jeonbuk National University Hospital from January to December 2019 within a week from acute ischemic stroke. Among all screened patients, five patients diagnosed as non-stroke diseases and 87 patients previously diagnosed with depressive mood disorder were excluded, as well as 25 additional patients who could not perform Hamilton Depression Rating Scale (HRDS) tests due to neurological deficiencies. A total of 227 patients were included in the final analysis (Figure 1). We conducted two studies designed in detail for the patients. Study Design 1: We analyzed the influential factors for PSD diagnosis after 3 months with no relation to the existence of PSD diagnosis during hospitalization (excluding the patients diagnosed with depression prior to hospitalization). Study Design 2: We analyzed the influential factors for improvements in PSD after 3 months, for patients that had confirmed PSD diagnosis at time of hospitalization (Supplementary Figure S1). We recorded age, sex, and body mass index (BMI). Blood pressure, diabetes, and atrial fibrillation, which are risk factors for cardiovascular disease, were also collected. Etiology of stroke was also surveyed according to the Trial of ORG 10,172 in Acute Stroke Treatment (TOAST) classification. The National Institute of Health Stroke Scale (NIHSS) was also evaluated three months after the stroke and at discharge. The Modified Rankin Scale (mRS) was also evaluated three months after discharge, for further neurological assessment. This study was conducted with the approval of the institutional review board of Jeonbuk National University Hospital (IRB No.: 2021-09-043).

### 2.2. Neuropsychiatric Test

We used HDRS to assess patients’ depression level. HDRS is one of the most commonly used depression scales, and is also used to evaluate PSD. It consists of 17 questions, of which 9 items score a maximum of 4 points, and 8 items a maximum of 2 points. The test usually takes approximately 20–30 min. Severity assessment and changes in depressive symptoms are the main indications provided by HDRS. HDRS is sensitive to changes in severe depression patients, as it emphasizes the somatization symptoms of depression. However, symptoms such as hypersomnia and hyperphagia, which are non-typical symptoms, are harder to assess. The HDRS rating scale is as follows: normal (0–6 points), mild (7–17 points), moderate (18–24 points), and severe depression (≥25 points). The total score ranges from 0 to 50 points, and a higher score indicates a worse depression level. Subjects were administered the HDRS test at admission and three months later (twice in total). Patients scoring 0–7 points were classified as normal (PSD-negative), while patients scoring ≥8 points were classified as PSD-positive.

### 2.3. Diagnosis of Acute Ischemic Stroke

We analyzed data of patients who visited the hospital within three days from the onset of neurological deterioration. In brain MRI, low signal intensity in the apparent diffusion coefficient (ADC) map and high signal intensity in the diffusion weighted image (DWI) is defined as an acute ischemic lesion. All imaging interpretations were independently conducted by two neurologists (CHL and HGK). In the case of disagreement, a third researcher (BSS) was called to decide.

### 2.4. Classification of Ischemic Stroke

All patients were classified into one of the following five subcategories, according to TOAST classification: large artery atherosclerosis (LAA), small vessel occlusion (SVO), cardioembolism (CE), stroke of other etiology (SOE), and stroke of undetermined etiology (SUE). For LAA, MRI shows a >1.5 cm lesion, and >50% stenosis in the parent artery. For CE, MRI shows a ≥1.5 cm lesion, same as LAA, with <50% stenosis in the parent artery. In addition, causes for high-risk CE are identified through electrocardiogram, 24-h Holter monitoring, or echocardiography. In case moderate risk factors are found, such as patent foramen ovale, patients are not classified as CE. SVO MRI shows a ≤1.5 cm lesion in penetrating arterial region with lacunar syndrome, with no prominent stenosis of proximal arteries. SUE has three subtypes: negative evaluation (cryptogenic), two or more causes identified, and incomplete evaluation. If rare causes such as hypercoagulable states or vasculitis are detected, the patient is categorized as SOE.

### 2.5. Statistical Analysis

Subjects were classified into two groups according to HRDS scores obtained three months after hospitalization (PSD-negative and PSD-positive group). Demographic information, clinical and laboratory information, and neurological severity were compared between the two groups. Continuous variables were analyzed using the Student T-test, and nominal variables were analyzed using Pearson’s chi-squared test. After three months, binomial logistic regression analysis was performed to determine factors affecting depression. Next, further analysis was conducted on the PSD-positive group. They were divided into two groups depending on whether depression had improved or not after three months (PSD-relieved and PSD-unrelieved group). The latter analysis was performed in the same way as the former analysis. All statistical analyses were performed using SPSS 25.0 (IBM Corp. Armonk, NY, USA).

## 3. Results

### 3.1. Factors Affecting PSD Three Months after Ischemic Stroke Occurrence

Among the 227 patients included, 163 (mean age, 67.1 ± 12.4 years) did not show PSD (PSD-negative group), while 64 (mean age, 68.2 ± 13.7 years) developed PSD (PSD-positive group) three months after the attack. The HDRS score of the PSD-positive group during admission was higher than that of the PSD-negative group (13.6 ± 9.7 versus 5.8 ± 4.3; *p* < 0.001). Moreover, the hospitalization period of the PSD-positive group was longer than that of the PSD-negative group (9.6 ± 4.9 days versus 7.8 ± 4.5 days; *p* = 0.010). The NIHSS score, mRS, and neurological measures were significantly higher in the PSD-positive group than in the PSD-negative group. There were no significant differences between the two groups with respect to the other clinical and laboratory data (Table 1). We used multiple logistic regression analysis to evaluate the factors influencing PSD after three months (Table 2). The results confirmed that HDRS score at admission (adjusted odds ratio (aOR) 1.22, 95% confidence interval (CI) 1.14–1.31; *p* < 0.001) and hospitalization period (aOR 1.11, 95% CI 1.02–1.20; *p* = 0.013) were significant factors.

### 3.2. Factors Impacting the Improvement of PSD

We conducted an additional analysis of the 109 patients diagnosed with depression at admission. These patients were divided into two groups—PSD-relieved group and PSD-unrelieved group—depending on the depression status three months after the stroke attack. The PSD-relieved group comprised 64 patients (mean age, 69.1 ± 12.2 years), while the PSD-unrelieved group comprised 45 patients (mean age, 68.8 ± 12.1 years). The HDRS score of the PSD-relieved group was significantly lower than that of the PSD-unrelieved group during admission (10.2 ± 3.4 versus 17.6 ± 8.9; *p* < 0.001). Although the hospitalization period of the PSD-unrelieved group was longer than that of the PSD-relieved group, the difference was not significant (9.4 ± 4.2 days versus 7.9 ± 4.3 days; *p* = 0.07). The NIHSS and mRS scores three months after stroke were significantly higher in the PSD-unrelieved patients (Table 3). No other significant differences were found between the two groups.

We performed multiple logistic regression analysis to identify the factors affecting the improvement of PSD after three months (Table 4). The results showed that the NIHSS score at discharge (aOR 0.80, 95% CI 0.68–0.94; *p* = 0.006) and HDRS score at admission (aOR 0.80, 95% CI 0.71–0.89; *p* < 0.001) were relevant factors for depression improvement after three months.

## 4. Discussion

We revealed the prevalence of PSD related to the HDRS score at admission and the hospitalization period. The possibility of PSD improvement was associated with the neurological severity at discharge and HDRS score at admission. 

PSD is the most common neuropsychiatric complication that can occur in stroke patients, and its prevalence is between 28% and 35% [11,12]. The World Health Organization reported that 15 million people worldwide suffered from stroke, indicating that the socioeconomic burden due to PSD could be extremely high [11,12]. Patients with PSD show more prominent functional and cognitive impairments, lower quality of life, and higher mortality one year after stroke than those without PSD [13,14]. Previous studies revealed that approximately 55% of patients with PSD experienced depression after cerebrovascular injury, and that they had not only more suicidal thoughts, but also less desire to live than patients without PSD. Consequently, PSD is to be considered a major factor interfering with patient recovery. Thus, PSD is the key criterion used to predict poor prognosis after stroke [14].

The pathophysiological mechanism of PSD remains unclear, but it is believed that complex interactions between various internal and external factors trigger PSD [15]. The hypotheses proposed so far can be divided into two major groups: (1) physiological changes in neurotransmitters, and (2) psychological and social influence [14]. In terms of neurotransmitters, low monoamine and brain-derived neurotrophic factor (BDNF) levels, hypothalamic–pituitary–adrenal dysregulation, and glutamate-mediated excitotoxicity have been suggested as the factors affecting PSD [14]. In terms of neuroanatomy, according to this hypothesis, the frontal lobe, hippocampus, limbic regions, and basal ganglia are associated with PSD development. The association between neurotransmitters and PSD has not yet been clearly demonstrated. The results of previous studies on the association between a reduction in BDNF and PSD development are contradictory [16]. Among seven studies investigating the association between BDNF and the pathogenesis of PSD, five confirmed a significant association between them, but two studies could not. Additionally, even the five studies that confirmed the association observed the association only in the early stage of stroke in most cases [16]. From the psychological and social perspectives, depression occurs due to physical discomfort and maladaptation to it [17,18], and the patient’s sex is also associated with the development of PSD [19].

Sex, insomnia, experience with psychiatric disorders, age, poor life experiences, living alone, the location of the brain lesion, NIHSS score, and Barthel index score have been suggested as the key risk factors influencing PSD [12]. In this study, we initially screened ischemic stroke patients for depression using HDRS; patients included in the study did not have depression at the onset of the stroke. We also conducted a follow-up HDRS assessment after three months. After additional analysis, we found that there was no significant difference in sex and age between the two groups after three months. We re-analyzed the data including only patients aged between 65 and 75, considering the relatively wide age distribution of the target group. However, age was still not a significant factor (Supplementary Tables S1 and S2). In previous studies, these two factors were identified as risk factors, although with conflicting results [17]. Since contradictory results have been reported [20], further studies are required to address this issue.

The PSD-positive group patients were hospitalized for a longer duration than the PSD-negative group patients. Moreover, the neurological severity of the PSD-positive group was higher than that of the PSD-negative group in all cases. We believe that higher neurological severity caused the PSD-positive group to have a longer hospitalization period. The results of the regression analysis showed that the hospitalization period independently was a significant factor in the development of PSD, but neurological severity was not. Hospitalization can deteriorate a patient’s mood, quality of life, and depressive status [21]. Moreover, depression has been reported to prolong the hospitalization period [22,23]. These results suggest that depression and hospitalization period may be correlated. As depression and hospitalization adversely influence each other, it is necessary to diagnose PSD as soon as possible and treat it appropriately. Appropriate psychosocial intervention in the early stages of stroke can increase the patient’s therapeutic compliance and shorten the hospitalization period [24,25]. There are few reports that the environmental factors surrounding the patient affected PSD. In a follow-up study of 1,094 ischemic stroke patients, the prevalence of PSD at 1 year was significantly higher in the case of living alone [10]. The analyzed factor in our study, the ‘hospitalization period’, is a risk factor that occurs during hospitalization, and compared to ‘living alone’, it can be said that the possibility of medical intervention is higher compared to the ‘living alone’ factor.

The HDRS scores of the PSD-positive group at admission were significantly higher than those of the PSD-negative group (*p* < 0.001). The mean HDRS score at admission of the PSD-positive group was 13.6 (95% CI 13.6 ± 9.7), indicating a mild level of depression. These results indicate that patients may develop depression from the early stage of stroke, but it is highly likely that the medical staff or caregivers might not recognize it because of its low severity. Thus, the possibility of early intervention to alleviate depression is less likely. The cumulative proportion of patients with PSD within five years from the onset of stroke is 39–52% [16].

This study showed that the NIHSS score at discharge and HDRS score at admission were factors significantly associated with the improvement of PSD after three months. The results indicated that PSD had a better outcome when the degree of neurological symptoms and depression was lower at the time of admission. Similarly, Kim et al. reported that depressive symptoms were better alleviated when the stroke symptoms were relieved, regardless of whether depression was treated [26]. Moreover, Ilut et al. revealed that a high NIHSS score at the time of admission was significantly associated with an increase in the incidence of PSD, and with prolonged recovery time from PSD after discharge [27].

We conducted analysis to confirm the association between PSD and laboratory findings. However, no significant laboratory factors were found. Previous studies reported that uric acid or C-reactive protein were associated with PSD during hospitalization [28,29]. Although we analyzed laboratory factors related to PSD during hospitalization, no significant laboratory factor, including uric acid and C-reactive protein, was found. We believe that the heterogeneity of patients’ characteristics, differences in psychiatric tools, and test timing may have influenced these results. Particularly, although the glucose and BUN levels of the PSD-unrelieved group were high, they were not statistically significant factors in the logistic analyses. We believe that the one-time laboratory results performed at the time of admission could not be associated with patients’ long-term psychiatric conditions. Additional studies, such as serial laboratory monitoring, are needed to address the association between PSD and laboratory data.

This study had several limitations. First, it considered only acute stroke patients. Therefore, it could not evaluate the possibility that depressive patterns may differ depending on the stage of the stroke. Second, as it was a retrospective study, and the sample was obtained from one single tertiary hospital, there could be a selection bias. Third, other methods used for diagnosing PSD were not considered. It was difficult to evaluate the atypical symptoms of depression, such as hypersomnia and hyperphagia, because this study assessed the presence of PSD in patients only through the HDRS test. Fourth, the follow-up period for assessing the patients’ depression severity was only three months, which is a relatively short period. Lastly, patients with a history of depression were excluded from the analysis, which made it impossible to determine the relationship between history of depression and PSD. 

## 5. Conclusions

Although PSD is a multifactorial and common post-stroke complication, it is treatable. The results of this study revealed that the risk of PSD increased when the HDRS score at admission was high and the hospitalization period was long. Moreover, chances of PSD improvement increased when the neurological severity at discharge and HDRS score at admission were low. When treating stroke patients, it is necessary to consider the possibility of PSD development in high-risk patients. It is believed that subsequent studies on the factors affecting PSD will greatly contribute to better understanding the pathophysiology of PSD. In particular, the factors associated with the improvement of PSD are expected to be important clues in related follow-up studies and patient management for PSD recovery. 

## Figures and Tables

**Table 1:** Baseline Characteristics of the Study Population.

Variables	All (*n* = 227)	Post-Stroke Depression		*p*-Value
		Negative (*n* = 163)	Positive (*n* = 64)	
**Demographics**				
Age (years)		67.1 ± 12.4	68.2 ± 13.7	*0.548*
Sex, male (%)		97 (59.5)	30 (46.9)	*0.084*
Body mass index (kg/m^2^)		22.8 ± 4.8	23.9 ± 3.2	*0.077*
**Cardiovascular risk**				
Hypertension		90 (55.2)	35 (54.7)	*0.943*
Diabetes mellitus		66 (40.5)	23 (35.9)	*0.527*
Atrial fibrillation		31 (19.0)	9 (14.1)	*0.378*
Dyslipidemia		36 (22.1)	16 (25.0)	*0.638*
Previous stroke		25 (15.3)	8 (12.5)	*0.585*
Previous ischemic heart disease		13 (8.0)	7 (10.9)	*0.479*
Smoking		38 (23.3)	11 (17.2)	*0.313*
Alcohol consumption		43 (26.4)	12 (18.8)	*0.227*
**TOAST classification**				
Large-artery atherosclerosis		39 (23.9)	17 (26.6)	*0.587*
Cardioembolism		40 (24.5)	19 (29.7)	
Small-vessel occlusion		28 (17.2)	9 (14.1)	
Stroke of undetermined etiology		42 (25.8)	17 (26.6)	
Stroke of other determined etiology		14 (8.6)	2 (3.1)	
**Psychological test**				
Onset to test (days)		2.7 ± 1.7	2.6 ± 1.8	*0.731*
HDRS score at admission		5.8 ± 4.3	13.6 ± 9.7	*<0.001*
**Hospitalization factors**				
Onset to admission (h)		13.7 ± 18.4	15.7 ± 20.3	*0.468*
Hospitalization period (days)		7.8 ± 4.5	9.6 ± 4.9	*0.010*
**Neurological severity**				
Initial NIHSS score		4.0 ± 4.4	5.4 ± 5.2	*0.034*
NIHSS score at discharge		2.1 ± 2.2	5.5 ± 5.0	*<0.001*
NIHSS score after 3 months		1.2 ± 1.6	4.7 ± 5.1	*<0.001*
Initial mRS		2.0 ± 1.4	2.7 ± 1.6	*0.004*
mRS at discharge		1.4 ± 1.2	2.7 ± 1.6	*<0.001*
mRS after 3 months		0.9 ± 1.1	2.3 ± 1.7	*<0.001*
**Laboratory findings**				
White blood cells (103/µL)		7.9 ± 3.5	8.2 ± 2.8	*0.501*
Hemoglobin (g/dL)		14.0 ± 5.1	13.2 ± 2.0	*0.222*
Platelets (103/µL)		244.4 ± 67.3	245.5 ± 60.6	*0.905*
Protein (g/dL)		6.4 ± 0.5	6.5 ± 0.6	*0.149*
Albumin (g/dL)		3.8 ± 0.4	3.8 ± 0.4	*0.297*
Aspartate transaminase (U/L)		24.5 ± 13.9	25.3 ± 25.7	*0.771*
Alanine transaminase (U/L)		21.1 ± 16.7	18.5 ± 11.3	*0.262*
Alkaline phosphatase (U/L)		54.1 ± 14.9	58.7 ± 20.1	*0.111*
Glucose (mg/dL)		103.1 ± 48.2	113.4 ± 51.0	*0.157*
Blood urea nitrogen (mg/dL)		15.4 ± 8.7	16.6 ± 7.9	*0.311*
Creatinine (mg/dL)		1.0 ± 0.8	1.0 ± 0.3	*0.592*
Total cholesterol (mg/dL)		186.8 ± 159.6	180.0 ± 42.1	*0.744*
Triglycerides (mg/dL)		118.3 ± 78.6	114.0 ± 88.2	*0.722*
Uric acid (mg/dL)		6.1 ± 13.7	5.0 ± 2.3	*0.526*
High-density lipoprotein (mg/dL)		43.2 ± 10.5	44.4 ± 11.5	*0.435*
Low-density lipoprotein (mg/dL)		106.4 ± 37.0	113.8 ± 38.5	*0.179*
Calcium (mg/dL)		8.7 ± 0.5	8.8 ± 0.4	*0.376*
Sodium (mEq/L)		149.7 ± 125.4	140.8 ± 3.8	*0.571*
Potassium (mEq/L)		4.0 ± 0.4	4.0 ± 0.4	*0.534*
Erythrocyte sedimentation rate (mm/h)		13.9 ± 13.6	13.0 ± 13.7	*0.664*
High-sensitivity C-reactive protein (mg/dL)		0.8 ± 1.4	1.4 ± 2.8	*0.094*
Thyroid stimulating hormone (μIU/mL)		1.6 ± 1.4	1.6 ± 1.9	*0.774*
Free thyroxine (ng/dL)		1.2 ± 0.3	1.2 ± 0.4	*0.348*
Glomerular filtration rate (mL/min)		89.4 ± 27.1	91.3 ± 29.8	*0.645*

TOAST, Trial of Org 10,172 in Acute Stroke Treatment; HDRS, Hamilton Depression Rating Scale; NIHSS, National Institutes of Health Stroke Scale; mRS, modified Rankin score.

**Table 2:** Logistic Regression Analysis of Post-Stroke Depression After Three Months.

Variables	Univariate Analysis		Multivariate Analysis *	
	Crude OR (95% CI)	*p*-Value	Adjusted OR (95% CI)	*p*-Value
Sex	0.60 (0.34–1.08)	*0.086*		
BMI	1.08 (0.99–1.17)	*0.079*		
HDRS score at admission	1.21 (1.14–1.29)	*<0.001*	1.22 (1.14–1.31)	*<0.001*
Hospitalization period (days)	1.08 (1.01–1.15)	*0.016*	1.11 (1.02–1.20)	*0.013*
Initial NIHSS score	1.07 (1.00–1.13)	*0.037*		
hs-CRP	1.16 (1.01–1.34)	*0.039*		

* Adjusted for sex, BMI, HDRS score, hospitalization period, initial NIHSS score, and hs-CRP. OR, odds ratio; CI, confidence interval; BMI, body mass index; HDRS, Hamilton Depression Rating Scale; NIHSS, National Institutes of Health Stroke Scale; hs-CRP, high-sensitivity C-reactive protein.

**Table 3:** Baseline Characteristics of PSD-Relieved and -Unrelieved Patients After Three Months.

Variables	All (*n* = 109)	Improvement of PSD		*p*-Value
		PSD-Relieved Group (*n* = 64)	PSD-Unrelieved Group (*n* = 45)	
**Demographics**				
Age (years)		69.1 ± 12.2	68.8 ± 12.1	*0.899*
Sex, male (%)		36 (56.3)	20 (44.4)	*0.225*
Body mass index (kg/m^2^)		23.1 ± 3.0	23.5 ± 3.2	*0.488*
**Cardiovascular risk**				
Hypertension		32 (50.0)	25 (55.6)	*0.567*
Diabetes mellitus		24 (37.5)	20 (44.4)	*0.467*
Atrial fibrillation		9 (14.1)	8 (17.8)	*0.599*
Dyslipidemia		16 (25.0)	12 (26.7)	*0.845*
Previous stroke		11 (17.2)	3 (6.7)	*0.148*
Previous ischemic heart disease		4 (6.3)	5 (11.1)	*0.484*
Smoking		15 (23.4)	7 (15.6)	*0.313*
Alcohol consumption		15 (23.4)	7 (15.6)	*0.313*
**TOAST classification**				
Large-artery atherosclerosis		16 (25.0)	10 (22.2)	*0.941*
Cardioembolism		15 (23.4)	11 (24.4)	
Small-vessel occlusion		8 (12.5)	8 (17.8)	
Stroke of undetermined etiology		21 (32.8)	14 (31.1)	
Stroke of other determined etiology		4 (6.3)	2 (4.4)	
**Psychological test**				
Onset to test (days)		2.8 ± 1.9	2.5 ± 1.9	*0.515*
HDRS score at admission		10.2 ± 3.4	17.6 ± 8.9	*<0.001*
**Hospitalization factors**				
Onset to admission (h)		13.4 ± 15.4	15.1 ± 17.3	*0.593*
Hospitalization period (days)		7.9 ± 4.3	9.4 ± 4.2	*0.070*
**Neurological severity**				
Initial NIHSS score		4.3 ± 4.2	6.2 ± 5.8	*0.072*
NIHSS score at discharge		2.2 ± 2.3	5.8 ± 5.1	*<0.001*
NIHSS score after 3 months		1.4 ± 1.8	4.8 ± 5.3	*<0.001*
Initial mRS		2.3 ± 1.5	2.7 ± 1.7	*0.124*
mRS at discharge		1.6 ± 1.2	2.7 ± 1.6	*<0.001*
mRS after 3 months		1.0 ± 1.1	2.2 ± 1.8	*<0.001*
**Laboratory findings**				
White blood cells (103/µL)		8.5 ± 4.6	8.3 ± 2.8	*0.885*
Hemoglobin (g/dL)		13.1 ± 1.9	13.2 ± 2.0	*0.784*
Platelets (103/µL)		249.8 ± 71.0	254.2 ± 59.4	*0.727*
Protein (g/dL)		6.4 ± 0.5	6.5 ± 0.6	*0.371*
Albumin (g/dL)		3.8 ± 0.5	3.8 ± 0.4	*0.547*
Aspartate transaminase (U/L)		24.6 ± 13.0	26.2 ± 30.0	*0.694*
Alanine transaminase (U/L)		20.4 ± 16.1	18.7 ± 11.1	*0.538*
Alkaline phosphatase (U/L)		54.8 ± 15.4	58.4 ± 19.6	*0.297*
Glucose (mg/dL)		96.1 ± 32.0	117.4 ± 51.8	*0.009*
Blood urea nitrogen (mg/dL)		14.2 ± 4.3	16.9 ± 8.8	*0.037*
Creatinine (mg/dL)		0.9 ± 0.3	1.0 ± 0.4	*0.713*
Total cholesterol (mg/dL)		172.8 ± 45.4	182.2 ± 41.0	*0.275*
Triglycerides (mg/dL)		123.5 ± 98.1	116.6 ± 93.7	*0.717*
Uric acid (mg/dL)		5.1 ± 1.6	4.9 ± 2.5	*0.696*
High-density lipoprotein (mg/dL)		43.9 ± 11.3	44.3 ± 11.4	*0.859*
Low-density lipoprotein (mg/dL)		105.6 ± 41.8	113.0 ± 36.9	*0.342*
Calcium (mg/dL)		8.7 ± 0.5	8.8 ± 0.5	*0.189*
Sodium (mEq/L)		164.9 ± 200.0	140.7 ± 3.7	*0.419*
Potassium (mEq/L)		3.9 ± 0.3	4.0 ± 0.5	*0.198*
Erythrocyte sedimentation rate (mm/h)		16.9 ± 17.1	12.8 ± 12.6	*0.190*
High-sensitivity C-reactive protein (mg/dL)		0.9 ± 1.6	1.8 ± 3.2	*0.096*
Thyroid stimulating hormone (μIU/mL)		1.4 ± 1.1	1.4 ± 1.2	*0.992*
Free thyroxine (ng/dL)		1.2 ± 0.4	1.3 ± 0.5	*0.362*
Glomerular filtration rate (mL/min)		91.9 ± 30.0	91.1 ± 30.0	*0.894*

PSD, Post-stroke depression; TOAST, Trial of Org 10,172 in Acute Stroke Treatment; HDRS, Hamilton Depression Rating Scale; NIHSS, National Institutes of Health Stroke Scale; mRS, modified Rankin score.

**Table 4:** Logistic Regression Analysis of Factors Improving Post-stroke Depression After Three Months.

Variables	Univariate Analysis		Multivariate Analysis *	
	Crude OR (95% CI)	*p*-Value	Adjusted OR (95% CI)	*p*-Value
NIHSS score at discharge	0.77 (0.67–0.88)	*<0.001*	0.80 (0.68–0.94)	*0.006*
Hospitalization period (days)	1.09 (0.99–1.20)	*0.080*		
HDRS score at admission	0.77 (0.68–0.86)	*<0.001*	0.80 (0.71–0.89)	*<0.001*
Glucose	1.01 (1.00–1.02)	*0.017*		
BUN	1.08 (1.00–1.16)	*0.060*		
hs-CRP	1.17 (0.98–1.39)	*0.078*		

* Adjusted for NIHSS at discharge, hospitalization period, HDRS score, glucose, BUN, and hs-CRP. OR, odds ratio; CI, confidence interval; BMI, body mass index; HDRS, Hamilton Depression Rating Scale; NIHSS, National Institutes of Health Stroke Scale; BUN, blood urea nitrogen; hs-CRP, high-sensitivity C-reactive protein.

## Data Availability

We can provide raw data to editors upon appropriate request.

## Supplementary Materials

The following are available online at https://www.mdpi.com/article/10.3390/jpm11111178/s1. Figure S1. Detailed study flow chart showing the patient selection according to two types of study design, Table S1. Baseline characteristics of study population according to post-stroke depression (age 65–75), Table S2. Logistic regression analysis of post-stroke depression after 3 months (age 65–75).
