# Supplementary material for: Factors Affecting Post-Stroke Depression in Acute Ischemic Stroke Patients after 3 Months
Source: J Pers Med. 2021 Nov 11;11(11):1178. doi: 10.3390/jpm11111178 (PMC8618912; doi:10.3390/jpm11111178)
Supplement: Supplementary file 1 [file jpm-11-01178-s001.zip › jpm-1415169-supplementary.pdf]

Supplementary Table S1. Baseline characteristics of study population according to post-stroke depression (age 65-75)

Variables (n=119)	post-stroke depression		*p* value
	Negative (n=90)	Positive (n=29)	
**Demographics**			
Age (years)	65.4±6.5	66.2±7.0	*0.559*
Male (%)	58 (64.4)	16 (55.2)	*0.371*
BMI (kg/m^2)	23.3±3.8	24.5±3.0	*0.119*
**Cardiovascular risk**			
Hypertension	48 (53.3)	15 (51.7)	*0.880*
Diabetes mellitus	42 (46.7)	11 (37.9)	*0.410*
Atrial fibrillation	19 (21.1)	3 (10.3)	*0.274*
Dyslipidemia	22 (24.4)	6 (20.7)	*0.678*
Previous stroke	17 (18.9)	5 (17.2)	*0.842*
Previous IHD	8 (8.9)	3 (10.3)	*0.814*
Smoking	19 (21.1)	6 (20.7)	*0.961*
Alcohol consumption	27 (30.0)	8 (27.6)	*0.804*
**TOAST classification**			
LAA	26 (28.9)	10 (34.5)	
CE	17 (18.9)	6 (20.7)	
SVO	16 (17.8)	4 (13.8)	*0.965*
SUE	25 (27.8)	7 (24.1)	
SOE	6 (6.7)	2 (6.9)	
**Psychological test**			

Onset to test (day)	2.7±1.8	2.8±2.0	*0.795*
HRDS score at admission	5.8±4.4	15.5±10.3	*<0.001*
**Hospitalization factors**			
Onset to admission (hr)	13.7±18.9	13.5±18.3	*0.976*
Hospitalization period (day)	8.0±4.2	8.8±4.7	*0.388*
**Neurological Severity**			
Initial NIHSS	3.5±3.8	4.8±5.4	*0.223*
NIHSS at discharge	1.9±2.2	4.7±5.1	*0.008*
NIHSS after 3 months	1.1±1.7	4.0±5.1	*0.005*
Initial mRS	1.9±1.3	2.4±1.6	*0.125*
mRS at discharge	1.4±1.1	2.2±1.6	*0.014*
mRS after 3 months	0.8±1.1	1.8±1.7	*0.005*
**Laboratory findings**			
WBC (103/uL)	7.6±2.5	8.0±2.6	*0.389*
Hb (g/dL)	14.0±4.0	13.8±1.5	*0.773*
PLT (103/uL)	242.5±67.8	242.9±61.7	*0.976*
Protein (g/dL)	6.5±0.5	6.5±0.6	*0.488*
Albumin (g/dL)	3.8±0.4	3.9±0.3	*0.226*
AST (U/L)	24.8±14.3	29.3±37.6	*0.352*
ALT (U/L)	20.7±12.8	20.8±13.1	*0.984*
ALP (U/L)	55.4±15.4	60.0±18.6	*0.207*
Glucose (mg/dL)	104.8±46.4	112.7±44.8	*0.423*
BUN (mg/dL)	14.3±4.8	17.1±9.1	*0.038*
Creatinine (mg/dL)	0.9±0.3	0.9±0.3	*0.950*

Total Cholesterol (mg/dL)	203.0±210.1	183.3±43.4	*0.631*
Triglycerides (mg/dL)	122.5±89.1	115.0±62.4	*0.683*
Uric acid (mg/dL)	5.1±1.4	5.2±2.8	*0.868*
HDL (mg/dL)	43.8±10.8	43.9±9.6	*0.960*
LDL (mg/dL)	107.4±37.8	118.8±42.3	*0.172*
Ca (mg/dL)	8.8±0.4	8.8±0.4	*0.519*
Na (mEq/L)	139.8±3.0	141.1±4.1	*0.059*
K (mEq/L)	4.0±0.3	4.1±0.4	*0.279*
ESR (mm/hr)	12.8±9.8	13.5±10.2	*0.738*
hs-CRP (mg/dL)	0.7±1.3	2.1±3.6	*0.041*
TSH (μIU/ml)	1.6±1.5	1.8±2.5	*0.555*
fT4 (ng/dL)	1.2±0.3	1.3±0.5	*0.293*
GFR (mL/min)	91.8±27.1	92.8±30.0	*0.873*

Supplementary Table S2. Logistic regression analysis of post-stroke depression after 3 months (age 65-75)

Variables	Univariate analysis		Multivariate analysis*	
	Crude OR (95% CI)	*p* value	Adjusted OR (95% CI)	*p* value
HRDS score at admission	1.24 (1.13-1.36)	*<0.001*	1.26 (1.14-1.39)	*<0.001*
NIHSS at discharge	1.25 (1.10-1.43)	*0.001*		
hs-CRP	1.32 (1.08-1.63)	*0.008*		

*Adjusted for sex, HRDS score, NIHSS at discharge, and hs-CRP
